# Case Report: Case Series of Human *Plasmodium knowlesi* Infection on the Southern Border of Thailand

**DOI:** 10.4269/ajtmh.19-0063

**Published:** 2019-10-07

**Authors:** Sutharinee Ngernna, Nattawan Rachaphaew, Suwich Thammapalo, Pathomporn Prikchoo, Opart Kaewnah, Khajohnpong Manopwisedjaroen, Kanit Phumchuea, Chayanut Suansomjit, Wanlapa Roobsoong, Jetsumon Sattabongkot, Liwang Cui, Wang Nguitragool

**Affiliations:** 1 Department of Molecular Tropical Medicine and Genetics, Faculty of Tropical Medicine, Mahidol University, Bangkok, Thailand;; 2 Mahidol Vivax Research Unit, Faculty of Tropical Medicine, Mahidol University, Bangkok, Thailand;; 3 Office of Disease Prevention and Control Region 12 Songkhla, Department of Disease Control, Ministry of Public Health, Songkhla, Thailand;; 4 Vector-Borne Diseases Control Center 12.2, Songkhla, Department of Disease Control, Ministry of Public Health, Songkhla, Thailand;; 5 Department of Internal Medicine, Morsani College of Medicine, University of South Florida, Tampa, Florida

## Abstract

Although human infections of *Plasmodium knowlesi* have been found throughout Southeast Asia, most cases originated from Malaysian Borneo. In Thailand, *P. knowlesi* malaria was considered extremely rare. However, during October 2017–September 2018, there was a surge in the number of reported *P. knowlesi* cases. Here, a series of six cases of *P. knowlesi* malaria found during this period in Songkhla and Narathiwat provinces of southern Thailand are presented. All cases were confirmed by polymerase chain reaction. The unprecedented case number in the affected area is a warning sign of an increasing *P. knowlesi* burden in the south of Thailand.

## INTRODUCTION

*Plasmodium knowlesi* is increasingly recognized as a major cause of human malaria. The natural hosts of this parasite are the long-tailed and pig-tailed macaques of Southeast Asia.^1,2^ The transmission of *P. knowlesi* is generally considered to be from monkeys to humans through local anopheline vectors,^3–5^ with reported human-to-human transmissions confined to blood transfusion^6,7^ and experimental infection.^8^ The first documented natural infection of humans with *P. knowlesi* was in 1965 when a traveler acquired the parasite from a visit to Southeast Asia.^9^ More recently, in 2004 and 2008, a large number of naturally acquired *P. knowlesi* infections in humans were reported in Sarawak state of Malaysian Borneo. In addition to Malaysia, *P. knowlesi* infections have now been observed throughout Southeast Asia.^10–17^ A number of infections were also found in international travelers.^18–24^

In Thailand, a retrospective study of blood samples obtained from malaria patients in northwestern Tak Province during 1996 uncovered a case of mixed species infection of *Plasmodium vivax* and *P. knowlesi.*^25^ In 2000, a case of human *P. knowlesi* malaria infection was stated in Prachuap Khiri Khan Province.^16^ During October 2006 and September 2007, 10 *P. knowlesi* infections were identified by polymerase chain reaction (PCR) in 1,751 malaria patients.^17^ During October 2008 and September 2009, a survey identified 23 *P. knowlesi* infections in 3,446 patients from various parts of the country.^25^ A separate study provided evidence of *P. knowlesi* infection in two patients working near the Thai-Myanmar border in Ranong Province.^26^

Recently, *P. knowlesi* clinical cases were reported, for the first time, by the Thai National Malaria Control Program (NMCP), with the nationwide total of 23 cases during October 2017 –September 2018.^27^ Here, we present six of these cases from Songkhla and Narathiwat provinces of Southern Thailand.

## METHODS

### Sample collection and malarial DNA extraction.

Six blood samples were collected from malaria patients who sought treatment at the Vector-Borne Disease Control Center (VBDC) in Songkhla Province or at Naradhiwas Rajanagarindra Hospital in Narathiwat Province from November 2017 to April 2018. Thin and thick smears were prepared, stained with 10% Giemsa, and examined under a microscope. Genomic DNA was extracted from dried blood spots on filter paper or blood pellets and used in nested PCR and quantitative PCR (qPCR) to confirm the parasite species. This study received human use exemption (certificate no. MUTM-EXMPT 2018-009) by the Ethics Committee of the Faculty of Tropical Medicine, Mahidol University, Thailand.

### *Plasmodium* species identification.

Nested PCR targeting the 18S rRNA genes was performed to detect the *Plasmodium* species.^28^ The first round of nested PCR was performed with *Plasmodium* genus–specific outer primers (ACGATCAGATACCGTCGTAATCTT and GAACCCAAAGACTTTGATTTCTCAT, 0.4 µM each). The reaction was set using GoTaq^®^ Green Master Mix (Promega, Madison, WI) in a 25-µL reaction volume under thermocycling conditions 95°C for 20 seconds, 55°C for 30 seconds, and 72°C for 30 seconds. One microliter of 1:50 dilution of the first-round product was used as the template for the second round of PCR with the same forward primer (ACGATCAGATACCGTCGTAATCTT) and a reverse primer specific to each species (CAATCTAAAAGTCACCTCGAAAGATG for *Plasmodium falciparum*, CAATCTAAGAATAAACTCCGAGAGGAAA for *P. vivax*, ACTGAAGGAAGCAATCTAAGAAATTT for *Plasmodium ovale*, AAGGAAGCTATCTAAAAGAAACACTCAT for *Plasmodium malariae*, and CTGAAGGAAGCAATCTAAGAGTTC for *P. knowlesi*). The second PCR was set in a 25-µL reaction volume using GoTaq Green Master Mix with 0.4 µM concentration of each primer and thermocycling conditions 95°C for 20 seconds, 60°C for 30 seconds, and 72°C for 30 seconds. The final PCR products were analyzed on 2% agarose gel. Dye-terminator sequencing of the nested PCR products was performed in both directions using the nested PCR primers through commercial service (Macrogen, Seoul, Republic of Korea). Positive *P. knowlesi* detection was further confirmed by *P. knowlesi*–specific TagMan 18S qPCR which used primers (GTTAGCGAGAGCCACAAAAAAGCGAAT and ACTCAAAGTAACAAAATCTTCCATA, 0.6 µM each) and a probe (HEX–TGCTTTATGTGCGCATCCTCTACCTA-BFQ, 0.5 µM) with iTaq^™^ Universal Probes Supermix (Bio-Rad, Hercules, CA) and two-step thermocycling conditions 95°C for 15 seconds and 60°C for 60 seconds*.*^28^

## CASE PRESENTATIONS

**Figure 1.**
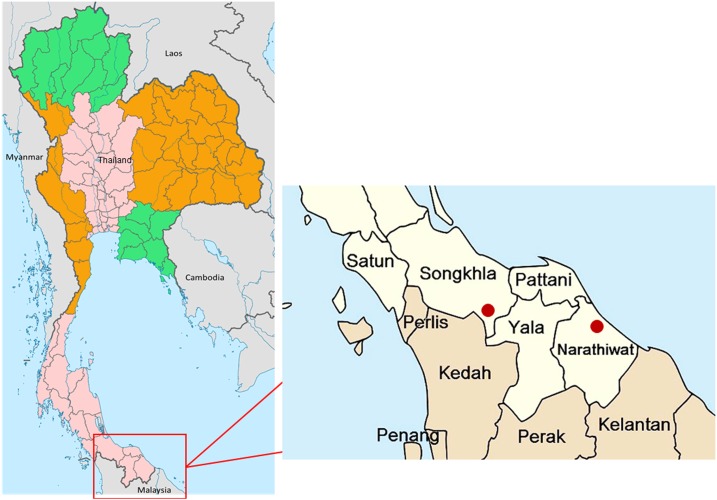
Map of Songkhla and Narathiwat provinces of Thailand and the Thai-Malaysian border. This figure appears in color at www.ajtmh.org.

**Figure 2.**
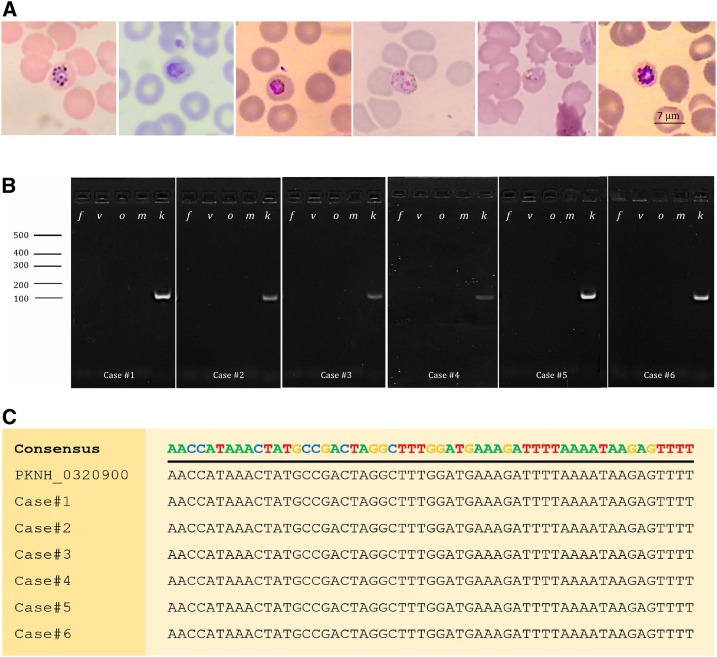
Diagnosis of Plasmodium *knowlesi* infection. (**A**) Light microscopic images of parasites with characteristics of *P. knowlesi* from Giemsa-stained thin blood smears. (**B**) Agarose gel images of nested polymerase chain reaction (PCR) demonstrating *P. knowlesi* infection using species-specific primers with the expected PCR product size of 110 base pairs. *f*, *v*, *m*, *o*, and *k* denote the species of parasites targeted by each PCR, from *Plasmodium falciparum*, *Plasmodium vivax*, *Plasmodium malariae*, *Plasmodium ovale*, and *P. knowlesi*. Numbers on the left indicate size in base pairs. (**C**) Sequences of the nested PCR products. The sequences of all six cases are identical to those of the reference *P. knowlesi* 18S rRNA gene PKNH_0320900 and differ from all known 18S rRNA genes of other human malaria parasites. This figure appears in color at www.ajtmh.org.

From November 2017 to April 2018, we identified six malaria patients with *P. knowlesi* infections, five from Songkhla Province and one from Narathiwat Province (Figure 1). These cases were suspected for *P. knowlesi* infection because of the unusual parasite morphology compared with more common *P. vivax* and *P. falciparum*. Blood films (Figure 2) of all cases showed trophozoites or schizonts without host cell enlargement, a feature consistent with *P. knowlesi* infection. Nested PCR, sequencing of the nested PCR products (Figure 2), and qPCR confirmed that infections were indeed due to *P. knowlesi* (Table 1).

The first case, a 22-year-old male resident of Na Thawi district, Songkhla Province, presented himself at the malaria clinic of the VBDC in Na Thawi in November 2017 with six days of fever, chills, and headache. The initial diagnosis by light microscopy was *P. vivax* malaria, and the patient was treated with chloroquine (25 mg/kg over 3 days) and primaquine (0.25 mg/kg/day for 14 days) and recovered fully. The patient had worked in a rubber plantation and camped out in the forest at Ban Na Kha, Malaysia. The camping cottage was surrounded by a wooded area populated by wild macaques, the potential reservoir of the parasite.

The second case, a 45-year-old man, presented at the Naradhiwas Rajanagarindra Hospital in Narathiwat Province in January 2018. He displayed symptoms including a 6-day history of daily fever, shivering, headache, nausea, vomiting, jaundice, pallor, and dark urine. His temperature during admittance to the hospital was 39.3°C. He did not have a history of either malaria or blood transfusion. A malaria rapid diagnosis test (RDT: SD BIOLINE Malaria Ag P.f/Pan test) was negative for *P. falciparum* histidine rich protein 2 antigen but positive for *Plasmodium* lactate dehydrogenase. The patient was considered to have *P. vivax* infection and treated with chloroquine (25 mg/kg over 3 days) and primaquine (0.25 mg/kg/day for 14 days) according to the standard *P. vivax* treatment. The patient reported to have worked in palm plantations and traveled to work in Garuntun, Malaysia, before becoming sick. His work place was near a damp rainforest inhabited by monkeys.

The third case was a 50-year-old man in Na Thawi district, Songkhla Province. He had fever for 9 days with a mild headache. He was initially diagnosed as having influenza, but did not recover after treatment with antiflu medication. The patient returned to seek medical aid at the malaria clinic of VBDC in Na Thawi in February 2018. After being diagnosed as having *P. vivax* infection by light microscopy, the patient was treated with chloroquine (25 mg/kg over 3 days) and primaquine (0.25 mg/kg/day for 14 days) and recovered fully. The patient was a rubber plantation worker and reported to have had camped out for 4 days in the forest at Baan Keun Nam, Kedah state of Malaysia.

The fourth case was a 48-year-old woman in Sadao district, Songkhla Province. The patient presented at the Sadao Hospital in April 2018 after having experienced fever, chills, abdominal pain, and severe headache for 4 days. The patient was initially diagnosed as having *P. vivax* malaria and cured by the standard chloroquine (25 mg/kg over 3 days) and primaquine (0.25 mg/kg/day for 14 days). The patient lived in a rubber plantation and was a rubber tapper, herdsman, and nontimber forest product finder. She frequently visited the forest along the Thai-Malaysian border.

The fifth case was a 32-year-old male resident of Sadao district, Songkhla Province. The patient presented at the malaria clinic of VBDC in Sadao in April 2018 with 5-day fever, chills, and severe headache. Initial diagnosis was *P. falciparum* malaria due to the predominance of ring-stage parasites in blood smears. The patient was cured by standard 3-day dihydroartemisinin (2.5 mg/kg/day)–piperaquine (20 mg/kg/day) treatment with a single dose of primaquine (30 mg) according to Thailand’s national guideline. The patient was a rubber plantation worker and nontimber forest product finder. He reported to have had regularly visited the forest inhabited by wild monkeys near Satun Province of Thailand.

The sixth case, also detected in April 2018, was a 35-year-old man in Saba Yoi district, Songkhla Province. The patient came to the malaria clinic of VBDC in Saba Yoi with 2-day fever and a mild headache. The patient was initially diagnosed by light microscopy as having *P. vivax* malaria and treated with chloroquine (25 mg/kg over 3 days) and primaquine (0.25 mg/kg/day for 14 days). The patient was a rubber plantation worker and wild animal hunter. He reported to have spent 4 days in a hilly forest at Baan Keun Nam, Kedah state of Malaysia, before becoming sick.

## DISCUSSION

We report a series of cases of *P. knowlesi* malaria in Songkhla and Narathiwat provinces of Southern Thailand. To our knowledge, this is the first report of *P. knowlesi* malaria in Songkhla Province, and the first from the Thai-Malaysian border area with blood smear evidence. The travel history of all patients revealed travel to an area inhabited by wild monkeys.

*Plasmodium knowlesi* infection is considered extremely uncommon in Thailand. During October 2017 and September 2018, a total of 10 cases of *P. knowlesi* malaria from the four border provinces (Songkhla, Yala, Narathiwat, and Satun) were reported for the first time by the Thai NMCP, despite the fact that the current national reporting system had been deployed since 2012. These cases forewarn the potential emerging threat of *P. knowlesi* in the southernmost area of Thailand. It is noteworthy that all reported *P. knowlesi* cases in this study were mistakenly diagnosed as *P. vivax* or *P. falciparum* during admission to the clinics or hospitals, suggesting that the hidden burden of knowlesi malaria might be much higher. It is important that blood smears of all suspected cases of *P. knowlesi* in Thailand and Peninsular Malaysia are confirmed by expert microcopy and molecular diagnosis to closely track the disease burden in the near future.

## Figures and Tables

**Table 1:** Summary of confirmed *Plasmodium knowlesi* malaria cases

Case no.	Age (years)	Gender	Ethnicity	Residence	Traveled to	Initial malaria diagnosis	Expert microscopy	PCR-based diagnosis		Date
								Nested PCR	Quantitative polymerase chain reaction	
1	22	M	Thai	Na Thawi, Songkhla	Baan Na Ka, Kedah state of Malaysia	*Plasmodium vivax*	*P. knowlesi*	*P. knowlesi*	*P. knowlesi*	November 2017
2	45	M	Thai	Jor I-rong, Narathiwat	Garuntun, Malaysia	*P. vivax*	*P. knowlesi*	*P. knowlesi*	*P. knowlesi*	January 2018
3	50	M	Thai	Na Thawi, Songkhla	Baan Keun Nam, Kedah state of Malaysia	*P. vivax*	*P. knowlesi*	*P. knowlesi*	*P. knowlesi*	February 2018
4	48	F	Thai	Sadao, Songkhla	Malaysian border	*P. vivax*	*P. knowlesi*	*P. knowlesi*	*P. knowlesi*	April 2018
5	32	M	Thai	Sadao, Songkhla	Satun, Thailand–Malaysian border	*Plasmodium falciparum*	*P. knowlesi*	*P. knowlesi*	*P. knowlesi*	April 2018
6	35	M	Thai	Saba Yoi, Songkhla	Baan Keun Nam, Kedah state of Malaysia	*P. vivax*	*P. knowlesi*	*P. knowlesi*	*P. knowlesi*	April 2018

F = female; M = male; PCR = polymerase chain reaction.
